# Antimicrobial Resistance in Chicken Meat: Comparing *Salmonella*, *Escherichia coli*, and *Enterococcus* from Conventional and Antibiotic-Free Productions

**DOI:** 10.3390/microorganisms13102227

**Published:** 2025-09-23

**Authors:** Camila Koutsodontis Cerqueira-Cézar, Aryele Nunes da Cruz Encide Sampaio, Evelyn Fernanda Flores Caron, Thaisy Tino Dellaqua, Lucas Franco Miranda Ribeiro, Leonardo Ereno Tadielo, José Carlos de Figueiredo Pantoja, Gustavo Guimarães Fernandes Viana, Gabriel Augusto Marques Rossi, Carlo Spanu, Fábio Sossai Possebon, Juliano Gonçalves Pereira

**Affiliations:** 1 Department of Animal Production and Preventive Veterinary Medicine, School of Veterinary Medicine and Animal Science (FMVZ), São Paulo State University (UNESP), Prof. Dr. Walter Mauricio Correa Street, Botucatu 18618-681, SP, Brazil; camila.cezar@unesp.br (C.K.C.-C.); aryele.sampaio@unesp.br (A.N.d.C.E.S.); evelyn.fernanda@unesp.br (E.F.F.C.); lucas.franco@unesp.br (L.F.M.R.); leonardoerenotadielo@gmail.com (L.E.T.); jose.pantoja@unesp.br (J.C.d.F.P.); gustavo.zero98@gmail.com (G.G.F.V.); fabio.possebon@unesp.br (F.S.P.); 2 Department of Structural and Functional Biology, Institute of Biosciences, São Paulo State University, Botucatu 18618-970, SP, Brazil; thaisy.dellaqua@unesp.br; 3 Department of Veterinary Medicine, University Vila Velha (UVV), Av. Comissário José Dantas de Melo, n.21, Vila Velha 29102-920, ES, Brazil; gabriel.rossi@uvv.br; 4 Department of Veterinary Medicine, University of Sassari (UNISS), 07100 Sassari, Italy; cspanu@uniss.it; 5 Institute for Biotechnology, São Paulo State University (UNESP), Tecomarias av, Botucatu 18607-440, SP, Brazil

**Keywords:** foodborne pathogens, food safety, multidrug resistance, poultry, one health

## Abstract

Chicken meat production is a critical component of the global protein supply, significantly influenced by rearing advancements, including the use of antimicrobial agents. However, the pervasive use of antibiotics has raised concerns regarding the occurrence of antimicrobial resistance (AMR). This study examined the prevalence and AMR profiles of *Salmonella* spp., *Escherichia coli*, and *Enterococcus* spp. in chicken meat from conventional and antibiotic-free (ABF) production chains. A total of 284 samples were analyzed for *Salmonella* spp. and *E. coli*, while 164 samples were tested for *Enterococcus* spp. From that, 143 were from conventional production chains and 141 were from ABF chains. The results indicated a 10.9% prevalence of *Salmonella* spp., 22.1% for *E. coli*, and 93.9% for *Enterococcus* spp. Regarding production chains, the conventional chain had 18.2% of the isolates for *Salmonella* spp., 20.3% for *E. coli*, and 91.6% for *Enterococcus* spp., while the ABF chain had 3.5% of the isolates for *Salmonella* spp., 24.1% for *E. coli*, and 96.3% for *Enterococcus* spp. In terms of AMR, 86.1% of the *Salmonella* spp. isolates that underwent the disk diffusion test were resistant to at least one antibiotic tested, 95.1% of *E. coli*, and 88.4% of *Enterococcus* spp. Notably, carbapenem resistance was detected in *Salmonella* spp., with 2.3% of isolates being resistant to imipenem, while resistance to vancomycin and linezolid was detected in *Enterococcus* spp., all of which are critically important antimicrobials. Comparisons between these production chains revealed significant differences in antibiotic resistance patterns in *Salmonella* spp. for two antibiotics, amoxicillin/clavulanic acid and nitrofurantoin, while no differences were observed in *E. coli*. For *Enterococcus* spp., resistance varied for three antibiotics: streptomycin, penicillin, and tetracycline. For all other antibiotics tested, the resistance profiles were consistent across both conventional and ABF production chains. Multidrug resistance (MDR) was observed in 90.7% of *Salmonella* spp. isolates, 42.9% of *E. coli* isolates, and 12.0% of *Enterococcus* spp. isolates. Statistically significant differences were noted in MDR prevalence between production chains, with conventional production systems exhibiting higher levels of MDR isolates compared to ABF systems. These findings underscore the need for targeted AMR control strategies that consider the complexity of resistance dynamics across production systems.

## 1. Introduction

The global poultry industry stands as a cornerstone of food security, providing accessible animal protein to billions of human beings while simultaneously confronting one of the modern world’s most pressing public health challenges: the occurrence of AMR. As poultry meat production continues to rise, projected to surpass 105 million tons annually by 2025, this sector’s dual role in nourishing populations and inadvertently propagating resistant microorganisms demands critical examination [1,2]. Within this context, Brazil emerges as a pivotal player, supplying over 33.0% of globally traded poultry while navigating the complex interplay between production efficiency and antimicrobial stewardship [1].

Poultry-derived meat offers unparalleled nutritional value, delivering essential amino acids, B vitamins, and minerals critical for human development [3]. However, the intensification of production systems has lead to widespread antimicrobial use for prophylaxis and treatment of diseases and to improve feed conversion, creating selective pressures that favor resistant bacterial populations [4]. Of particular concern are *Salmonella* spp., *Escherichia coli*, and *Enterococcus* spp., pathogens that dominate poultry microbiomes and serve as sources and transmitters of resistance genes [5,6,7].

Globally, the utilization of antimicrobials in conventional poultry systems administered for growth promotion and disease prevention stands at 148 milligrams per population correction unit (PCU), with Brazil positioned among the top antibiotic consumers in agriculture, trailing behind China and the United States [8,9]. Projections suggest a near doubling of antimicrobial usage by 2030 to meet the rising consumer demand [9]. Consumption of undercooked, contaminated meat, occupational exposure during poultry handling in farms or abattoirs, and environmental spread via poultry litter used as organic fertilizer are just some of the multiple ways antimicrobial-resistant bacteria can be transferred to humans [10,11,12,13].

Although the production cycle for broiler chickens in conventional systems is relatively short, typically around 42 days, and might suggest lower antibiotic usage compared to the longer rearing periods of cattle and swine, the high stocking density in poultry farming significantly increases the risk of disease transmission [14,15]. As a result, this dense environment often necessitates the use of antibiotics to control and prevent bacterial infections [16]. Moreover, antibiotic administration in these systems is typically performed at the herd or flock level, rather than at the individual animal level [17].

In response, antibiotic-free (ABF) production systems have gained traction, driven by consumer demand and regulatory shifts [18,19]. This shift mirrors growing consumer demand for products perceived as both safer and more environmentally responsible [20]. Transitioning to ABF requires multifaceted strategies: enhanced biosecurity protocols, nutritional interventions (prebiotics, organic acids), and vaccination programs to mitigate diseases [21]. This shift carries economic trade-offs: ABF systems face higher production costs due to increased mortality rates, extended grow-out periods, and greater resource use [19,22]. Recent studies demonstrate that even ABF systems harbor resistant lineages on poultry meat, suggesting the need for a more holistic change to production as well as antimicrobial withdrawal time [18,21,23,24,25].

In this scenario, this study explores AMR in *Salmonella* spp., *E. coli*, and *Enterococcus* spp. isolated from poultry meat originating from conventional and ABF production chains. By comparing resistance patterns across these systems, the research aims to evaluate the potential of ABF practices in curbing AMR while highlighting persistent risks to public health. Ultimately, this study seeks to support more informed decisions in poultry production, contributing to long-term strategies that reconcile food safety with responsible antimicrobial use.

## 2. Results

### 2.1. Overall

A total of 284 chicken meat samples were analyzed, including 143 from the conventional production chain and 141 from the ABF chain. The findings are summarized in Table 1. *Salmonella* spp. was detected in 31 samples, with a prevalence of 18.2% (26/143) in conventional products and 3.5% (5/141) in ABF products. From these positive samples, 195 isolates were confirmed: 177 from the conventional chain and 18 from the ABF chain. As shown in Table 1, *E. coli* was isolated from 63 samples (22.1%), including 29 from the conventional chain (20.3%) and 34 from the ABF chain (24.1%). Out of the 98 isolates detected, 47 were from the conventional chain and 51 were from the ABF chain (Table 1). Among the 164 samples analyzed for *Enterococcus* spp., 154 (93.9%) were positive, with 76 isolates (91.6%) from the conventional chain and 78 (96.3%) from the ABF chain (Table 1). In total, 146 isolates from the conventional chain and 153 isolates from the ABF samples (Table 1) were detected. Given the substantial number of *Enterococcus* spp. isolates, two isolates from each sample, when feasible, underwent antibiogram analysis.

As shown in Table 2, a total of 472 isolates (79.7%) exhibited resistance to at least one antibiotic. Of these, 316 (85.4%) originated from the conventional chain, corresponding to 104 positive samples (72.7%), while 156 (70.2%) came from the ABF chain, representing 82 samples (58.1%). The complete resistance profiles for all bacteria analyzed are presented in Table 3.

### 2.2. Resistance Profile Obtained in Salmonella spp. Isolates

The resistance profile of *Salmonella* spp. isolates, as detailed in Table 3, revealed notably high resistance to several commonly used antibiotics, particularly β-lactams, with 100.0% of ABF isolates and 75.7% of conventional isolates resistant to amoxicillin/clavulanic acid (AMC) and fluoroquinolones, such as ciprofloxacin, to which 84.2% of conventional isolates were resistant. In contrast, resistance to aminoglycosides, carbapenems, and macrolides was minimal or absent, indicating preserved susceptibility to these classes. Of the antibiotics with detected resistance, only AMC and nitrofurantoin (NIT) showed significant differences between the production chains, with higher resistance unexpectedly observed in ABF isolates (Table 3). A total of 182 isolates (93.3%) were classified as MDR, including 164 (92.7%) from the conventional chain and all 18 (100.0%) from the ABF chain, as shown in Table 4.

### 2.3. Resistance Profile Obtained in Escherichia coli Isolates

Following the *Salmonella* spp. findings, we evaluated the resistance profile of the *E. coli* isolates, which notably demonstrated resistance to 13 out of the 16 antibiotics tested (Table 3), with no significant differences between the resistance profiles of the two chicken production chains. High resistance rates (≥50.0%) to sulfamethoxazole/trimethoprim (SUT) were observed in both production systems. Additionally, notable resistance to ampicillin and tetracycline was observed in both chains, indicating the potential presence of resistance genes to these two long-established antibiotics. Resistance levels in both production chains were comparable.

Critical antibiotic classes, such as macrolides, aminoglycosides, and fluoroquinolones, exhibited lower rates of resistance in both chains (Table 3). Carbapenems maintained their efficacy, with both imipenem (IPM) and meropenem (MER) demonstrating no observed resistance. Azithromycin (AZI) also remained highly effective, with 98.0% of isolates being susceptible and only 2.0% showing resistance. Nitrofurantoin demonstrated substantial effectiveness, with 98.0% of total isolates being susceptible.

Among the 98 isolates tested with the disk diffusion method, 42 (42.9%) were identified as MDR, with 18 (38.2%) originating from the conventional production chain and 24 (47.0%) from the ABF chain (Table 4).

### 2.4. Resistance Profile Obtained from Enterococcus spp. Isolates

In *Enterococcus* spp. isolates, resistance to penicillin was detected only in the conventional chain (Table 3). Resistance to vancomycin, a high-priority critical item for human medicine [26], was observed in both production systems. Teicoplanin (TEI) resistance was low but present (2.1% for conventional chain and 0.7% for ABF chain). Gentamicin (GEN) resistance remained minimal across chains (2.1% and 5.2%), while streptomycin (EST) resistance was significantly higher in conventional isolates (11.0% to 2.6% in ABF). Resistance to tetracycline (TET) was also significantly greater in the conventional chain compared to the ABF chain (52.7% in conventional to 34.6% in ABF). Concerning MDR, among the 299 isolates tested with the disk diffusion method, only 32 (10.7%) were identified as MDR, with 22 (15.0%) originating from the conventional chain and 10 (6.5%) from the ABF chain (Table 4). Compared to *Salmonella* spp. and *E. coli*, *Enterococcus* spp. showed lower overall resistance levels.

### 2.5. Multidrug Resistance Profiles

The analysis of all three pathogens revealed the presence of MDR isolates across both conventional and ABF production chains, as shown in Table 4. Notably, the most prevalent resistance profiles observed in all pathogens tested were identical across both production chains (Table 5). The most common MDR profile of *Salmonella* spp. in conventional production was AMC-CTF-CIP-SUT-AMP-TET (42.9%), while the same profile appeared in 44.4% of ABF production isolates. The second most common pattern was AMC-CFO-CTF-CIP-SUT-AMP-TET for both chains, representing 10.7% of the conventional isolates and 22.0% of the ABF isolates. The most predominant MDR profile of *E. coli* both in conventional and ABF production was SUT-AMP-TET, representing 14.9% of the conventional isolates and 9.8% of the ABF isolates. The second most prevalent pattern was also the same for both chains. *Enterococcus* spp. had lower rates of MDR when compared to the other enterobacteria tested in this study but had the same profiles for both conventional and ABF chains, including TET-LNZ-CIP-VAN and LNZ-CIP-VAN (0.6% for conventional and 1.3% for ABF) and TET-LNZ-VAN (16.6%), while in ABF production, profiles such as TET-LNZ-CIP-VAN (16.6%) were prevalent. Although *Enterococcus* spp. exhibited lower overall MDR rates compared to the other pathogens (Table 4), it is important to emphasize that resistance was already present to both linezolid and vancomycin, which are critical antibiotics in human medicine.

**Figure 1:**
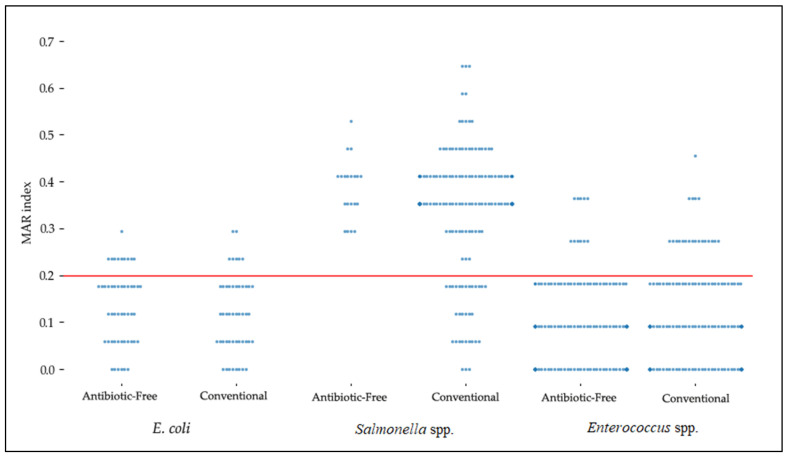
Scatter plot showing the MAR index of *E*. *coli*, *Salmonella* spp., and *Enterococcus* spp. from both production chains. Each point represents one isolate. Isolates above the 0.2 threshold are considered to originate from an elevated selective pressure environment.

The mean multiple antibiotic resistance (MAR) index across all isolates was 0.18, with 218 isolates (35.4%) presenting values above the 0.2 threshold. The scatter plot (Figure 1) provides an overview of the distribution of the MAR index across individual isolates, highlighting the differences between the production chain and bacteria. When analyzed by microorganism and production system, the average MAR index values were 0.12 for *E. coli* (0.13 in ABF and 0.12 in conventional isolates), 0.35 for *Salmonella* spp. (0.39 in ABF and 0.34 in conventional isolates), and 0.09 for *Enterococcus* spp. (0.09 in ABF and 0.11 in conventional isolates). Overall, *Salmonella* spp. displayed the highest MAR index values among the three bacterial groups, while *Enterococcus* spp. presented the lowest.

## 3. Discussion

This study evaluated AMR in *Salmonella* spp., *E. coli*, and *Enterococcus* spp. isolated from poultry meat sourced from conventional and ABF production chains. In the present study, *Salmonella* spp. was isolated from 10.8% of chicken meat samples, with a considerably higher prevalence in the conventional production chain compared to the ABF chain. Among the 195 isolates analyzed, 93.3% were classified as MDR, with no statistically significant difference between the two chains. The most common resistance profiles were identical in both systems, notably the AMC-CTF-CIP-SUT-AMP-TET pattern, indicating widespread dissemination of resistant strains throughout production.

These findings align with the results of Park et al. [5], who analyzed retail chicken meat and reported that 81.8% of *Salmonella* isolates were resistant to multiple β-lactams, such as ampicillin, cefazolin, and ceftazidime. Furthermore, 81.8% of isolates exhibited resistance to five or more antibiotics, including 93.7% of isolates from the ABF chain, reinforcing the absence of significant differences between systems, which is consistent with our observations.

Punchihewage-Don et al. [27] also documented a high frequency of tetracycline resistance in *Salmonella* spp. from organic and non-organic chickens. The authors reported over 60 distinct resistance profiles, many shared between production systems, as well as highly MDR isolates in both groups. This reflects the ability of these pathogens to maintain complex resistance patterns even in the absence of direct antimicrobial use.

Conversely, some studies have identified considerably lower resistance levels in ABF systems. Cui et al. [28], analyzing chicken embryos and environmental samples, reported an MDR rate of only 20.2% in ABF poultry compared to 93.5% in conventional poultry. Resistance to ampicillin, amoxicillin, ceftazidime, tetracycline, and other antimicrobials was also significantly lower in ABF poultry [28].

Another noteworthy finding was the detection of resistance, albeit at low levels, to carbapenems (such as imipenem), whose use is only allowed for human medicine and which are classified by the WHO as highest priority, critically important antimicrobials, along with macrolides (such as azithromycin), which are used in both human and veterinary medicine and are also designated as critically important antimicrobials [26].

In the present study, *E. coli* was isolated from 22.2% of the samples, with a similar distribution between conventional and ABF production chains. Among the 98 confirmed isolates, resistance was observed in 13 of the 16 antimicrobials tested, with the highest resistance rates to sulfamethoxazole–trimethoprim, ampicillin, and tetracycline. Although no statistical differences were observed between the production systems, slightly higher resistance to certain antibiotics, such as ciprofloxacin, gentamicin, and azithromycin, was noted in isolates from the ABF chain, despite broadly similar resistance profiles overall.

These findings align with the results of Tofani et al. [29], who analyzed cecal content from broilers and reported resistance rates ≥50.0% to ampicillin, sulfonamides, and tetracycline, irrespective of the production system. Pesciaroli et al. [30], while evaluating cecal content after slaughter, also documented high resistance to these antimicrobials and found that the odds of resistance to β-lactams, sulfonamides, and quinolones were approximately 50.0% lower in ABF poultry compared to conventional systems. Nevertheless, the authors detected resistance in both systems, similar to our study, suggesting that discontinuing antibiotic use does not fully eliminate resistant bacteria.

Retail chicken meat studies corroborate these trends. Sanchez et al. [31] observed no statistical difference in *E. coli* resistance to ampicillin or erythromycin between conventional and “No Antibiotics” products. Conversely, Davis et al. [32], while assessing chicken meat sold in the U.S., concluded that production practices had minimal influence on resistance prevalence, although gentamicin resistance was higher in conventional isolates, a contrast to our findings, where this resistance was marginally elevated in the ABF chain, albeit without statistical significance.

In this study, *Enterococcus* spp. exhibited the highest prevalence among the three pathogens evaluated, isolated from 98.7% of the samples. Although the frequency of resistance to multiple antimicrobial classes was lower compared to the other microorganisms, 10.7% of isolates were classified as MDR. Resistance rates to streptomycin and tetracycline were significantly higher in isolates from the conventional production chain than in those from the ABF chain. The presence of resistance to vancomycin and linezolid in both systems, albeit at low frequencies, is concerning given the role of linezolid as a last-resort drug for enterococcal infection, especially in cases caused by vancomycin-resistant *Enterococcus* [33]. Linezolid resistance has been linked to genes such as *optr*A, *poxt*A and *cfr*, all of which have been detected in poultry isolates [34,35]. The transferability of these genes, combined with indirect selective antimicrobial pressure from the use of linezolid in human clinical settings [36], may reach poultry production via environmental or other routes, which could explain the resistance in some of our *Enterococcus* spp. isolates.

Our findings are supported by Kilonzo-Nthenge et al. [37], who analyzed components of retail-sold conventional and organic chicken carcasses. The authors observed higher resistance in isolates from conventional poultry, particularly to streptomycin and penicillin. In ABF poultry, while streptomycin resistance was also elevated, resistance frequencies for most tested antibiotics were considerably lower, especially for β-lactams and macrolides.

Kim et al. [7] did not observe a statistically significant difference in AMR between conventional and organic chicken carcasses; however, MDR isolates were more prevalent in conventional chickens, which is consistent with the findings of the present study. Zhang et al. [38] identified high overall resistance frequencies in both systems (over 75.0% of isolates resistant to at least one antimicrobial), demonstrating that resistance in *Enterococcus* spp. may persist regardless of direct antibiotic exposure during production.

The MDR profiles of *Salmonella* spp., *E. coli*, and *Enterococcus* spp. reveal a concerning landscape of AMR across both conventional and ABF production chains. *Salmonella* spp. leads with the highest frequency of MDR (93.3%), with the predominant profile (AMC-CTF-CIP-SUT-AMP-TET) present in both chains at similar proportions. As tetracyclines have been used in veterinary medicine as growth promoters since 1950 [39] and sulfonamides were the first antibiotics used in veterinary medicine at therapeutic doses [40], a higher resistance profile was already expected in our isolates. However, the presence of resistance to critical antibiotics, such as macrolides and carbapenems in *Enterobacteriaceae* and vancomycin and linezolid in *Enterococcus* spp., is particularly alarming. These antibiotics are listed by the WHO as crucial for human medicine [26], underscoring the gravity of AMR in these pathogens [41], especially given the high consumption of poultry meat and the potential for resistant bacteria to persist throughout the production and handling process. These organisms may reach consumers through undercooked meat [42], cross-contamination in domestic kitchens [43], or environmental exposure, with a significant risk of horizontal gene transfer to clinically relevant human pathogens [24]. Interestingly, although MDR rates were higher in *Salmonella* spp. and *E. coli*, the resistance profiles did not show statistically significant differences between production chains. This highlights that there may not be a real difference in AMR between the two systems for these bacteria, despite the absence of antibiotic use in ABF chains.

In our study, the overall mean MAR index was 0.18, with more than one-third of the isolates showing values above 0.2, indicating exposure to high-risk antibiotic contamination sources [44]. The mean MAR index for *E. coli* was 0.12, similar to the value reported in Egypt (0.16) [45]. It also falls within the lower end of the ranges described in Sri Lanka (0.1–0.8) [46] and Bangladesh (0.14–1.00 [47], while remaining below the higher range of 0.38–1.00 observed by Tanzin et al. [48] in the same country. These results suggest that while the resistance levels in our isolates were lower than those documented in South Asia, resistant *E. coli* populations remain widespread in poultry meat. For *Salmonella* spp., our MAR index values (0.34–0.39) were higher than those reported in India, where Rawat et al. [49] found averages of 0.22–0.24 in ABF and conventional isolates, respectively. Similarly, Karim et al. [50] reported that 68.8% of *Salmonella* isolates presented MAR values above 0.2, which is consistent with the elevated indices observed in our study (84.5%). In contrast, *Enterococcus* spp. displayed much lower MAR index values (0.09), reinforcing their comparatively lower resistance burden in poultry meat. Taken together, these findings highlight that although MAR index values vary considerably across geographic regions and production systems, the high MAR indices observed in *Salmonella* spp. in our study underscore their role as critical reservoirs of MDR in poultry production.

Several factors may explain why AMR did not significantly decrease in ABF samples. For instance, day-old chicks may acquire resistant bacteria in hatcheries or during transport, potentially undermining efforts within the production chain to control the spread of AMR [6]. Cross-contamination during slaughter, where ABF and conventional animals may not be processed separately, might contribute to resistance spread [51]. Another reason may be explained by the persistence of resistance genes and bacteria in the farm environment due to previous antimicrobial use, as well as the capacity of certain MDR clones to survive and adapt to environmental stresses, enabling their continued presence even after antibiotics have been withdrawn [52,53]. Compounding this risk, poultry litter, a common organic fertilizer, serves as a reservoir for resistant bacteria, facilitating environmental dissemination of AMR genes into soil and water systems [54].

Overall, the findings of this study confirm the widespread presence of antimicrobial-resistant and MDR bacteria in retail poultry, regardless of production method. While ABF systems reduce direct antibiotic exposure, they do not eliminate the presence of resistant strains [18]. Our findings challenge the perception that ABF labeling necessarily reflects lower AMR risk and underscore the need for stricter oversight and upstream interventions beyond antibiotic exclusion. This illustrates the need for a holistic approach to antimicrobial stewardship that includes not only on-farm practices but also slaughterhouse hygiene, environmental controls, and robust surveillance programs throughout the production chain.

The comparison of MDR profiles across studies presents challenges due to differences in the types of antibiotics tested and the concentrations of disks used in disk diffusion assays, which highlights a limitation in current methodologies. An additional limitation is the lack of *Enterococcus* species-level identification, as certain *Enterococcus* species, such as *E. gallinarium* and *E. casseliflavus*, exhibit intrinsic resistance to vancomycin [55]; therefore, we cannot differentiate between intrinsic and acquired resistance. Another limitation lies in the variability of sample types used across the literature. Our study focused on retail meat samples, which represent the final step of the production chain and are therefore subject to multiple potential sources of contamination, including transport, carcass handling, and processing. In contrast, other studies analyze cloacal swabs [6], litter [56], or carcasses [57], which have not been exposed to all possible stages where contamination with antimicrobial-resistant bacteria may occur. These inconsistencies can hinder accurate cross-study comparisons and the assessment of resistance patterns on a broader scale. Therefore, standardized approaches to antimicrobial susceptibility testing are warranted to enable more robust and reliable evaluations of MDR profiles. Additionally, the detection of identical MDR profiles in both production systems reinforces the need for comprehensive and continuous surveillance, as well as targeted intervention strategies, to better understand and control the spread of AMR.

In Brazil, AMR in poultry has been investigated at earlier stages of the production chain, such as at the slaughterhouse level [58] and in frozen chicken carcasses available in retail markets [23,59]. However, to our knowledge, this is the first study to evaluate phenotypic AMR in *Salmonella* spp., *E. coli*, and *Enterococcus* spp. isolated directly from retail chicken meat cuts in the country. By targeting this final step of the production chain, our study provides a more realistic picture of the resistance profiles reaching consumers, accounting for potential contamination during slaughter, processing, transport, and retail handling.

Future research should prioritize mapping the points of contamination with resistant bacteria across the entire poultry production chain in antibiotic-free systems. Longitudinal studies, from hatcheries to retail, are essential to identify where and how resistant strains are introduced or maintained. This knowledge is critical for designing targeted interventions that can truly differentiate between ABF products in terms of microbial safety. As consumer expectations and market value are often higher for ABF poultry, understanding and reducing hidden AMR risks are both public health priorities and commercial necessities. Future studies should aim to incorporate MIC-based methodologies or explore zone diameter epidemiological cut-off values (ECOFFs), where available, to more accurately monitor shifts in resistance phenotypes and better characterize the impact of antimicrobial use in poultry environments.

## 4. Materials and Methods

### 4.1. Sampling

Sample collecting was carried out at various supermarkets in the city of Botucatu, São Paulo, Brazil. The 284 analyzed samples consisted of chicken cuts, which were categorized into two distinct groups: conventional (both frozen and chilled samples) and ABF (only frozen samples). A total of 143 chicken cuts were collected from the conventional production chain, while 141 cuts were obtained from the ABF chain (with a certification seal on the label). To avoid sample duplication, care was taken to collect cuts from different batches. All samples were stored in polystyrene foam containers to preserve their temperature and were transported to the lab facility. There, they were held in cold storage at 4 °C until processed within 24 h. Each sample analyzed comprised 25 g of broiler chicken, and each of these samples was then specifically designated for the isolation of pathogens.

### 4.2. Salmonella spp. Detection

The *Salmonella* spp. investigation was conducted in accordance with the ISO 6579 guidelines [60]. The samples (25 g) underwent an enrichment process with 225 mL of buffered peptone water (BPW) and were subsequently incubated at 37 °C for 18–20 h. Following incubation, 1 mL of the enrichment was transferred to the tetrathionate broth (TT), while 0.1 mL was transferred to the Rappaport-Vassilidis Soya broth (RVS). The broths followed incubation at specific temperatures, TT being incubated at 37 °C for 24 h and RVS incubated at 41.5 °C for 24 h. Subsequently, an aliquot was inoculated onto xylose lysine deoxycholate and bismuth sulfite agar and incubated at 37 °C for 24 h. Two to five characteristic colonies (black-centered colonies on red agar, indicating H2S production and lysine decarboxylation) from each sample were submitted for biochemical and serological evaluations for presumptive identification. Successively, the isolates were submitted to molecular analyses for species confirmation.

### 4.3. Escherichia coli Detection

Each 25 g sample was enriched with 225 mL of BPW, followed by homogenization for 3 min and incubation at 37 °C for a period of 18–24 h. After this step, aliquots of the broth were inoculated onto MacConkey agar and incubated at 37 °C for 18–24 h. The resulting plates were examined, and three colonies exhibiting typical characteristics of *E. coli* (pink colonies on MacConkey agar, indicating lactose fermentation) were selected per sample. These suspected colonies were then transferred to Eosine Methilene Blue (Emb Levine) agar and incubated at 37 °C for 18–24 h. Two to three characteristic colonies from each sample were collected and stored in BHI broth at −20 °C and submitted to molecular confirmation.

### 4.4. Enterococcus spp. Detection

The investigation of *Enterococcus* spp. followed the protocol proposed by Klein et al. [61], with modifications. Due to the markedly higher recovery rate of *Enterococcus* spp. compared to *Salmonella* spp. and *E. coli*, we limited the number of *Enterococcus* samples analyzed to prevent disproportionate representation and allow for more balanced comparisons across the three bacterial groups. For the 164 samples analyzed for *Enterococcus* spp., an enrichment process was initiated with 225 mL of BPW broth, followed by homogenization for 3 min and then incubation at 37 °C for a period of 18–24 h. After incubation, the samples were inoculated onto BBL Enterococcosel™ agar (Bile Esculin Azide Agar) (Oxoid Ltd., Basingstoke, UK) and kept in incubation for 48 h at 37 °C. Three to five characteristic colonies (black colonies on EnterococcelTM agar, indicating esculin hydrolysis) from each sample were collected and stored in BHI broth at −20 °C and submitted to molecular confirmation.

### 4.5. Molecular Confirmation of Species

All isolates obtained from characteristic colonies underwent molecular identification utilizing PCR. DNA extraction was conducted in-house in all instances through thermal lysis. For the DNA extraction process, the methodology outlined by Peres et al. [62] was employed for *Enterococcus* spp., and the protocols by Pui et al. [63] and Dias et al. [64] were employed for *E. coli* and *Salmonella* spp., respectively.

The *invA* gene (Table 6) was employed for the identification of all *Salmonella* spp. isolates using conventional PCR techniques. Analogously, indicative colonies of *E. coli* underwent selection for molecular validation through the *uspA* gene (Table 6). For isolates suspected to be *Enterococcus* spp., molecular analysis was executed via the exploration of the *tuf* gene (Table 6) for genus identification. For the last pathogen, due to the high number of isolates, confirmation was limited to a single isolate per sample.

### 4.6. Antimicrobial Resistance Profile

The confirmed isolates underwent antibiotic resistance testing across different antibiotic classes by the Kirby–Bauer disk diffusion method. Antibiotic selection for testing followed the guidelines outlined by CLSI, 2020 [68]. For microorganisms belonging to the *Enterobacteriaceae* family (*Salmonella* spp. and *E. coli*), 16 antibiotics were assessed, including amoxicillin+clavulanate (10 µg), aztreonam (30 µg), gentamicin (10 µg), streptomycin (10 µg), imipenem (10 µg), meropenem (10 µg), ceftiofur (30 µg), cefoxitin (30 µg), ciprofloxacin (5 µg), norfloxacin (10 µg), trimethoprim-sulfamethoxazole (25 µg), azithromycin (15 µg), ampicillin (10 µg), chloramphenicol (30 µg), tetracycline (30 µg), and nitrofurantoin (300UI). For *Enterococcus* spp. isolates, 10 antibiotics were selected, including ampicillin (10 µg), penicillin (10 µg), vancomycin (30 µg), teicoplanin (30 µg), ciprofloxacin (5 µg), chloramphenicol (30 µg), linezolid (30 µg), tetracycline (30 µg), gentamicin (120 µg), and streptomycin (300 µg), with the last two administered at high doses to test isolates for high aminoglycoside resistance.

The isolates were cultured in BHI broth and incubated at 37 °C for 18–24 h. Following this step, the cultures were diluted in BHI until reaching turbidity equivalent to that of a 0.5 MacFarland tube, as read on the DensiCHECK^TM^ Plus equipment (bioMérieux, Durham, NC, USA). The bacterial suspension was then inoculated onto Mueller–Hinton agar plates, followed by the addition of the antibiotic disks to be tested. The sets were then incubated at 37 °C for 18–24 h. At the end of this period, the inhibition zone was measured, and the results were classified as resistant, intermediate, or sensitive, following CLSI guidelines, 2020 [68]. Isolates demonstrating resistance to three or more groups of antibiotics tested were categorized as MDR [69]. For classification purposes, a method was used that defined samples as resistant to antimicrobials by accounting for both intermediate and full resistance, ensuring a more comprehensive assessment of AMR. The MAR index of the isolates was determined as a/b, where ‘a’ represents the number of multiple antibiotics to which the specific isolates are resistant, and ‘b’ represents the number of multiple antibiotics to which the specific isolates are exposed [44].

### 4.7. Data Analysis

The prevalence of *Salmonella* spp., *E. coli*, and *Enterococcus* spp. in meat cuts from conventional and ABF was analyzed using the chi-square test of independence with the PROC FREQ function in SAS 9.4 (SAS Institute Inc., Cary, NC, USA) and a 95.0% confidence interval. When fewer than five counts were observed in at least 25.0% of the cells, Fisher’s exact test was also performed with a 95.0% confidence interval. Similarly, the prevalence of AMR for each antimicrobial was compared between organic and conventional processing using the chi-square test of independence. A significant level of 5.0% was adopted in all tests.

## 5. Conclusions

This study demonstrates the widespread occurrence of antimicrobial-resistant and MDR bacteria, including *Salmonella* spp., *E. coli*, and *Enterococcus* spp., in poultry meat from both conventional and ABF production chains. While ABF systems aim to reduce antimicrobial exposure, the detection of similar resistance profiles in both chains highlights that removing antibiotics from production alone is not sufficient to eliminate resistant strains. The presence of resistance to critically important antimicrobials, such as carbapenems, vancomycin, and linezolid, emphasizes the urgent need for surveillance and control strategies that encompass the entire production chain, from hatcheries to retail. These findings reinforce the importance of adopting a One Health approach to antimicrobial stewardship, integrating biosecurity, environmental management, and food safety measures to mitigate the dissemination of resistance from animal production to humans.

## Figures and Tables

**Table 1:** Comparative prevalence of *Salmonella* spp., *Escherichia coli*, and *Enterococcus* spp. and confirmed isolate counts in conventional versus ABF retail poultry meat.

Chain	*Salmonella* spp.			*E. coli*			*Enterococcus* spp.		
	N (%)	*p* Value	Isolates	N (%)	*p* Value	Isolates	N (%)	*p* Value	Isolates
Conventional	26/143 (18.2%)	0.0001	177	29/143 (20.3%)	0.4767	47	76/83 (91.6%)	0.3287	146
ABF	5/141 (3.5%)		18	34/141 (24.1%)		51	78/81 (96.3%)		153
Total	31/284 (10.9%)		195	63/284 (22.2%)		98	154/164 (93.9%)		299

ABF (antibiotic-free); *p* = *p*-values indicate the statistical significance of differences between conventional and ABF chains. *p* ≤ 0.05 denotes a significant difference. Confidence interval: 95.0%.

**Table 2:** Number and percentage of resistant isolates and positive chicken meat samples from conventional and ABF production chains. Percentages are calculated within each chain.

Chain	Resistant Isolates (n/N, % Within Chain)	Positive Samples (n/N, % Within Chain)
Conventional	316/370 (85.4%)	104/143 (72.7%)
ABF	156/222 (72.7%)	82/141 (58.1%)
Total	472/592 (79.7%)	186/284 (65.5%)

ABF (antibiotic-free).

**Table 3:** Comparative analysis of antibiotic resistance frequency (%) between isolates from conventional and ABF production chains for *Salmonella* spp., *Escherichia coli*, and *Enterococcus* spp.

Antibiotic		*Salmonella* spp.		*E coli*		*Enterococcus* spp.	
		CON.	ABF	CON.	ABF	CON.	ABF
Aminoglycosides	GEN	0.0	0.0	4.3	8.0	2.1	5.2
	EST	9.0	0.0	6.4	6.0	11.0	2.6 ^a^*
Folate pathway antagonist	SUT	91.0	100.0	57.4	50.0	-	-
Beta-lactams	AMC	75.7	100.0 ^a^	0.0	6.0	-	-
Carbapenems	IPM	2.3	0.0	0.0	0.0	-	-
	MER	0.0	0.0	0.0	0.0	-	-
Cephalosporins	CFO	22.6	27.8	0.0	8.0	-	-
	CTF	84.8	100.0	8.5	16.0	-	-
Phenicoles	CLO	7.3	0.0	6.4	8.0	1.4	0.7
Fluoroquinolones and Quinolones	CIP	84.2	83.3	2.1	4.0	25.3	23.5
	NOR	0.0	0.0	0.0	0.0	-	-
Glycopeptides	VAN	-	-	-	-	20.5	23.5
	TEI	-	-	-	-	2.1	0.7
Macrolides	AZI	1.7	0.0	0.0	2.0	-	-
Monobactams	ATM	5.1	0.0	0.0	4.0	-	-
Nitrofurans	NIT	7.9	27.8 ^a^	4.3	0.0	-	-
Oxazolidinones	LNZ	-	-	-	-	7.5	7.2
Penicillins	AMP	90.4	100.0	63.8	62.0	2.7	0.0
	PEN	-	-	-	-	3.4	0.0 ^a^
Tetracyclines	TET	85.3	100.0	44.6	42.0	52.7	34.6 ^a^

* Rows marked with the superscript letter “^a^” indicate that the differences observed had a *p*-value < 0.05. Resistance rates include isolates classified as resistant and intermediate. CON (conventional production chain); ABF (antibiotic-free production chain); AMC (amoxicillin/clavulanic acid); AMP (ampicillin); ATM (aztreonam); AZI (azitromycin); CFO (ceftiofur); CIP (ciprofloxacin); CLO (cloramphenicol); CTF (cefoxitin); EST (streptomycin); GEN (gentamycin); IPM (imipenem); LNZ (linezolid); MER (meropenem); NIT (nitrofurantoin); NOR (norfloxacin); PEN (penicillin); SUT (sulfamethoxazole/trimethoprim); TEI (teicoplanin); TET (tetracycline); VAN (vancomycin). Confidence interval: 95.0%.

**Table 4:** Prevalence and total number of MDR isolates of *Salmonella* spp., *Escherichia coli*, and *Enterococcus* spp. by production chain.

Bacteria	Total MDR	Conventional	ABF
*Salmonella* spp.	182/195 (93.3%)	164/177 (92.7%)	18/18 (100.0%)
*Escherichia coli*	42/98 (42.9%)	18/47 (38.2%)	24/51 (47.0%)
*Enterococcus* spp.	32/299 (10.7%)	22/147 (15.0%)	10/153 (6.5%)
Total	256/592 (43.2%)	204/371 (55.0%) ^a^	52/222 (23.4%) ^b^

MDR (multidrug-resistant); ABF (antibiotic-free); Superscript ‘^a^’ and ‘^b^’ denotes a statistically significant difference in the prevalence of MDR isolates between conventional and ABF chains (*p* < 0.001). Confidence interval: 95.0%.

**Table 5:** MDR and their distribution among isolates of *Salmonella* spp., *E. coli*, and *Enterococcus* spp. in ABF and conventional production chains.

Pathogens	MDR Profiles from ABF	Frequency ABF	MDR Profiles from Conventional	Frequency Conventional
*Salmonella* spp.	AMC-CTF-CIP-SUT-AMP-TET	8 (44.4%)	AMC-CTF-CIP-SUT-AMP-TET	76 (42.9%)
	AMC-CFO-CTF-CIP-SUT-AMP-TET	4 (22.0%)	AMC-CFO-CTF-CIP-SUT-AMP-TET	19 (10.7%)
*E coli*	SUT-AMP-TET	5 (9.8%)	SUT-AMP-TET	7 (14.9%)
	SUT-AMP-CLO-TET	3 (5.9%)	SUT-AMP-CLO-TET	1 (2.1%)
*Enterococcus* spp.	TET-LNZ-CIP-VAN	2 (1.3%)	TET-LNZ-CIP-VAN	1 (0.6%)
	LNZ-CIP-VAN	2 (1.3%)	LNZ-CIP-VAN	1 (0.6%)

MDR (multidrug-resistant); ABF (antibiotic-free); AMC (amoxicillin/clavulanic acid); AMP (ampicillin); CIP (ciprofloxacin); CLO (cloramphenicol); CTF (cefoxitin); LNZ (linezolid); NIT (nitrofurantoin); SUT (sulfamethoxazole/trimethoprim); TET (tetracycline); VAN (vancomycin). Confidence interval: 95.0%.

**Table 6:** Target genes employed for the molecular confirmation of *Salmonella* spp., *E. coli*, and *Enterococcus* spp. isolates in poultry.

Pathogen	Target Genes	Sequence (5′-3′)	Bp	Reference
*Salmonella* spp.	*invA*	F: TTGTTACGGCTATTTTGACCA R: CTGACTGCTACCTTGCTGATG	521	[65]
*Escherichia coli*	*uspA*	F: CCGATACGCTGCCAATCAGT R: ACGCAGACCGTAGGCCAGAT	884	[66]
*Enterococcus* spp.	*tuf*	F: TACTGACAAACCATTCATGATG R: AACTTCGTCACCAACGCGAAC	112	[67]

## Data Availability

The original contributions presented in this study are included in the article. Further inquiries can be directed to the corresponding author.
